# Comprehensive review of the evidence regarding the effectiveness of community–based primary health care in improving maternal, neonatal and child health: 5. equity effects for neonates and children

**DOI:** 10.7189/jogh.07.010905

**Published:** 2017-06

**Authors:** Meike Schleiff, Richard Kumapley, Paul A Freeman, Sundeep Gupta, Bahie M Rassekh, Henry B Perry

**Affiliations:** 1 Department of International Health, Johns Hopkins Bloomberg School of Public Health, Baltimore, Maryland, USA; 2 UNICEF, New York, New York, USA; 3 Independent consultant, Seattle, Washington, USA; 4 Department of Global Health, University of Washington, Seattle, Washington, USA; 5 Medical epidemiologist, Lusaka, Zambia; 6 The World Bank, Washington, District of Columbia, USA

## Abstract

**Background:**

The degree to which investments in health programs improve the health of the most disadvantaged segments of the population—where utilization of health services and health status is often the worst—is a growing concern throughout the world. Therefore, questions about the degree to which community–based primary health care (CBPHC) can or actually does improve utilization of health services and the health status of the most disadvantaged children in a population is an important one.

**Methods:**

Using a database containing information about the assessment of 548 interventions, projects or programs (referred to collectively as projects) that used CBPHC to improve child health, we extracted evidence related to equity from a sub–set of 42 projects, identified through a multi–step process, that included an equity analysis. We organized our findings conceptually around a logical framework matrix.

**Results:**

Our analysis indicates that these CBPHC projects, all of which implemented child health interventions, achieved equitable effects. The vast majority (87%) of the 82 equity measurements carried out and reported for these 42 projects demonstrated “pro–equitable” or “equitable” effects, meaning that the project’s equity indicator(s) improved to the same degree or more in the disadvantaged segments of the project population as in the more advantaged segments. Most (78%) of the all the measured equity effects were “pro–equitable,” meaning that the equity criterion improved more in the most disadvantaged segment of the project population than in the other segments of the population.

**Conclusions:**

Based on the observation that CBPHC projects commonly provide services that are readily accessible to the entire project population and that even often reach down to all households, such projects are inherently likely to be more equitable than projects that strengthen services only at facilities, where utilization diminishes greatly with one’s distance away. The decentralization of services and attention to and tracking of metrics across all phases of project implementation with attention to the underserved, as can be done in CBPHC projects, are important for reducing inequities in countries with a high burden of child mortality. Strengthening CBPHC is a necessary strategy for reducing inequities in child health and for achieving universal coverage of essential services for children.

Martin Luther King, Jr., in a speech in 1966 to the Medical Committee for Human Rights, proclaimed, “*Of all the forms of inequality, injustice in health care is the most shocking and inhumane*” [1]. Between countries and within countries, inequalities in health status are by and large considered inequitable because they can be greatly reduced or even eliminated through stronger health programs. In spite of marked improvements in health programming and health status around the world, inequities are not diminishing as much as many countries and stakeholders had hoped [2–4]. Particularly since the 1990s, measuring and working to reduce inequities — with a goal ultimately to reach zero — has been on the global health agenda from global and national policy–makers to major donors [3–6].

Issues of health inequities for maternal, neonatal and child health (MNCH) in low– and middle–income countries (LMICs) are being increasingly studied. Some progress is being made in a number of areas such as the use of insecticide–treated net (ITN) usage to prevent malaria, exclusive breastfeeding, and immunization coverage [7]. Further, approaches for reaching underserved populations are receiving increasing attention in order to achieve the Millennium Development Goals (MDGs) [4] and the newly established Sustainable Development Goals (SDGs) [8]. At the global level, a recent declaration [9] brought together national public health associations from around the world to focus and mobilize action for achieving health equity by building evidence, addressing the social determinants of health (SDH), and incorporating equity components into health policies. Nonetheless, a great deal of learning and work remains to be done in order to accelerate reductions in health inequities.

Recent evidence from tracking of the “Countdown to 2015” [7–12], when the MDGs were supposed to be achieved, shows that population coverage of key interventions provided by health services is improving for the poorest quintiles of national populations at a rate faster than that for the wealthiest quintiles. However, the poorest quintiles are still facing markedly lower levels of coverage than the wealthier quintiles in most Countdown countries (the 74 countries with 97% of the world’s child and maternal deaths, ie, the greatest burden of maternal, neonatal and child mortality). Even though some measures of health inequities are slowly improving, substantial challenges remain for how to accelerate this progress [3,4]. The gaps are wider for interventions that require access to fixed health facilities or repeat contacts with a health provider (such as a skilled birth attendant) than for interventions that can be delivered through outreach strategies at the community level [5]. The countries that have made rapid progress in coverage are those that effectively reached the poorest families [5]. This is despite starting with great inequities. For example, in Cambodia and Sierra Leone in 2000 the richest had much higher coverage than the rest, but by 2014 this difference had disappeared [13].

The terminology around inequities, inequalities, and disparities has been the topic of debate over the past decades [14]. We will use the following interpretations of the terms in the context of this article. *Disparities* and *inequalities* (often used interchangeably) refer to differences among socially or geographically defined groups in health service utilization, in risk factors for unfavorable health outcomes, in levels of morbidity or mortality (collectively referred to here as health status) – essentially encompassing the entirety of epidemiological inquiry [14]. *Inequity*, however, “does not refer generically to all differences in health, but focuses specifically on the sub–set of differences that are ‘avoidable, unfair, and unjust” [14]. In practice, studies of inequities in health often focus on the degree to which marginalized and disadvantaged groups within geographically defined populations have less access to health care resources and have lower utilization of health care services.

Such differences stem from characteristics such as educational level, income (or wealth), race, child’s gender, geographic location, religion, or other characteristics of a social group that persistently produce social barriers that can lead to health outcomes that are different from those of other social groups. Beyond the semantics, Braveman argues that how we define and use these terms has important and relevant implications for policy and practice, and these definitions can determine the measures used to determine progress and even the flow of funding for different interventions [14]. Alternately, Taylor suggested a definition of equity as the, “distribution of benefits according to demonstrated need [health status] rather than on the basis of political or socioeconomic privilege” [15]. He focused on equity of the health status of populations rather than more proximal indicators of health system inputs or health service utilization.

From a public health perspective, it is important to examine the equity of both health program implementation and health outcomes among different socially and geographically defined sub–populations. Overall improvements in the health of a population can occur without every sub–group benefiting equally [7,16,17].

The equity effects of MNCH programs have undergone perhaps the greatest scrutiny of any global health program. One of the recent drivers for this scrutiny was the challenge of meeting the MDGs by 2015 and accelerating progress in countries that were lagging behind [11,12,18]. Analysts observed that, within many countries, inequities in child mortality were widening in spite of overall downward trends in child mortality [19].

Analyses have been conducted using Demographic and Health Survey (DHS) and Multiple Indicator Survey (MICS) data from MDG Countdown Countries regarding the population coverage of key maternal and child health interventions by income quintiles to assess equity in coverage [4,5]. Results showed trends toward increased equity in coverage of key interventions. Some of the most equitably implemented interventions are those that can fairly easily be implemented within communities, such as ITN utilization, promotion of exclusive breastfeeding (EBF), and community–based provision of immunizations [7,10,20,21]. At the same time, widening inequities were observed among different population sub–groups for interventions that require facility–based, higher–level personnel such as skilled birth attendants and treatment of serious childhood illness [22]. These interventions often require a more developed health system including education and support of skilled personnel, more advanced equipment, referral processes, and other support structures in order to be effective, and thus tend to be less evenly distributed among population groups [7,10].

While equity issues are often considered from a national or large–population perspective, they may exist at the local level as well. In one long–standing comprehensive health program in Haiti serving 148 000 people with a strong community–based service delivery system, the utilization of health facilities, the population coverage of key interventions, and the health outcomes of sub–groups of the program area differed markedly among those living in the more isolated mountain communities compared to those is nearer valley communities. This reality persisted despite great efforts being made to extend both primary health care services and access to CHWs equally throughout the program area [23].

This article makes two contributions to the equity literature. First, it consolidates for the first time the evidence regarding the equity effects of CBPHC programs on child health and organizes them around a logical framework. Second, this article reviews the various dimensions of equity that child health programs need to consider, including wealth (or household assets), maternal education, child’s sex, geographic location, and gender of the child’s caregiver and identifies dimensions where limited analysis has been conducted.

## METHODS

### Data sources

We used a recently assembled database containing assessments of 548 studies, projects or programs (referred to collectively as projects) that used CBPHC (defined in the initial paper in this series [24]) to improve neonatal or child health (henceforth referred to as child health) and to document these improvements. In brief, CBPHC was considered to be one or more interventions carried out in the community outside of a health facility. The additional presence of one or more facility–based interventions did not disqualify the project from inclusion.

The database and its assembly have been described elsewhere in this series [24]. In short, peer–reviewed documents, reports and books assessing the impact of one or more CBPHC interventions on child health (coverage of a key child survival indicator, nutritional status, serious morbidity, or mortality) in LMIC settings, among children in a geographically defined population, were selected. Two independent data extraction reviews were carried out and followed by an independent consolidated summative review. Data from the latter review were transferred to electronic database.

From this database, we identified a sub–set of 42 projects that had carried out an equity analysis as part of their assessment using the process described in the following section.

### Article review and inclusion process

Using the PRISMA guidelines for systematic reviews on health equity [25,26], we identified a sub–set of 138 articles in which equity was mentioned in one or more of the following fields in the CBPHC project database: 1) the title of the article, 2) the documentation of the process of the intervention, 3) part of the data analysis strategy, or 4) in the notes provided by the reviewers of the assessment for inclusion in the systematic review. We carefully reviewed this sub–set of equity–relevant assessments and excluded assessments in which equity was not actually analyzed across population subgroups. After this focusing phase, we were left with 43 projects to examine further.

**Figure 1:**
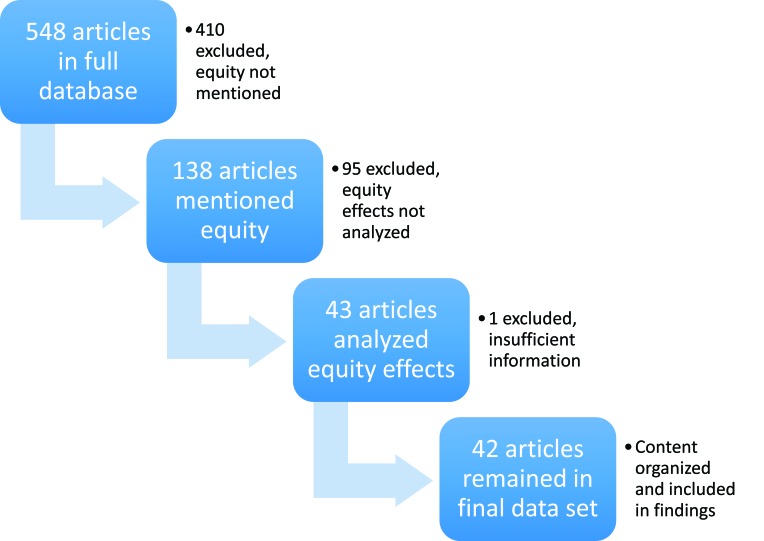
Overview of sequence of article review and inclusion/exclusion criteria.

Two of the authors (MS and RK) separately reviewed each of these 43 projects and extracted additional data on how equity was defined in each assessment, what data sources were utilized for assessment of equity effects, and what the outcome on equity actually was. The metrics from each project being assessed were stratified into log–frame categories (input, process, output, outcome, impact). One article was excluded from the analysis because it did not provide sufficient information on how equity was analyzed, leaving 42 articles in the final data set (**Figure 1**). Aside from the availability of adequate information on equity analysis in each article, the quality of the study was not assessed.

### Criteria for equity analysis

In order to identify the diverse criteria utilized among the studies to analyze equity, we created open–text responses as we reviewed each assessment, and then categorized them into common themes as we identified commonalities among the identified categories. We summarize the categories below and provide examples for some of the less–common categories. In our literature review, we identified a USAID report [27] on incorporating equity into project designs for MNCH that offered guidance on identifying disadvantaged groups that should be considered in implementing equitable MNCH projects. The USAID report referred to these groups by the acronym PROGRESS (**P**lace of residence, **R**ace, **O**ccupation, **G**ender, **R**eligion, **E**ducation, and **S**ocioeconomic **S**tatus) [28]. This typology provided guidance for the kinds of characteristics to look for and how to organize the findings from the reports we analyzed.

### Categorization of equity outcomes

We created three categories of outcomes for the various equity indicators used by the assessments included in our analysis (pro–equitable, equitable, and inequitable, as defined in **Box 1**). We categorized indicators as pro–equitable if findings favored underserved populations and were statistically significant or, if tests of statistical significance were not carried out, the study authors described their results as having practical significance. Indicators with findings that were similar for underserved groups as for the other groups were categorized as equitable. Indicators with findings that showed unfavorable outcomes for underserved populations were categorized as inequitable.

Box 1 **Definitions**

**Pro–equity effect:** when inputs, processes, and outcomes for disadvantaged groups improved more than for advantaged groups by the end of project implementation.

**Equity effect:** when inputs, processes, and outcomes for disadvantaged groups improved to the same degree as advantaged groups by the end of project implementation.

**Inequity effect:** when inputs, processes and outcomes for disadvantaged groups improve less than for advantaged groups by the end of project implementation.

**Dimension of equity:** A characteristic — such as household income, level of maternal education, or whether a child lives in an urban or rural areas — that can be used to compare population groups through an equity lens and determine whether different sub–groups of the population receive different levels of services or achieve different outcomes.

**Equity indicator:** An indicator of child health—such as rates of home visitation for newborns, for example — that was analyzed across a dimension of health equity.

These categories helped us to differentiate between several important equity outcomes –namely when disadvantaged sub–groups were benefitting less, equally, or more than other sub–groups. If disadvantaged groups were benefitting less, this was an inequitable outcome. When disadvantaged groups were benefitting equally, this was noted as a good sign, though not a fully optimal outcome since disadvantaged groups often need to make additional progress in order to overcome inequities.

### Organization of identified metrics for health equity into a logical framework

**Figure 2:**
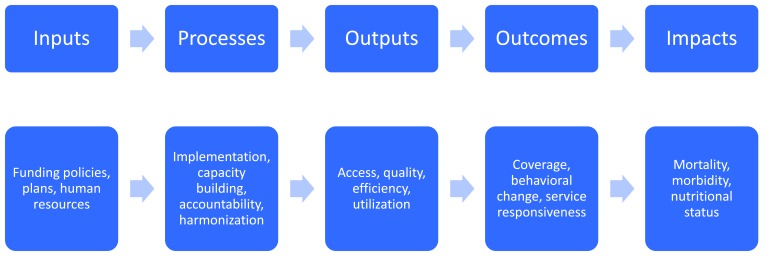
Generalized log frame for health projects.

Barros et al. [19] offer a framework for analysis of health equity from the standpoint of an individual person’s experience with an illness, beginning with the socioeconomic context through exposures to disease, vulnerability to succumbing to disease, and the outcomes and consequences of illness. While this approach helped us think through the various ways that equity can influence child health work, we opted to organize the indicators of health equity used by the assessments included in our analysis by utilizing a different framework of analysis from the standpoint of project implementation: beginning with inputs and processes, and then moving to outputs, outcomes, and impacts [29] to track at what point in project implementation equity dimensions were assessed. This made it possible to identify gaps and opportunities from a project planning and implementation perspective. **Figure 2** below provides a graphic representation of the conceptual flow of this log–frame matrix from one phase to another.

We created a matrix for each phase of the logical framework and, for each of the included indicators, tabulated the equity effects of each project. For each cell of the matrix, we described the content of the project and drew conclusions from the available evidence.

Each assessment was further analyzed to determine the criteria used to define equity, the type of data used to assess equity, and the scope of the assessment as well as the types of indicators measured in the assessment. The definition of equity was not pre–determined, and the definitions of equity used in the assessments were categorized after the list of equity indicators used in the projects had been reviewed. This was done to avoid missing any relevant equity indicators that might not have fit into a pre–determined definition of equity.

The type of data used to assess equity was defined as primary or secondary. The term primary data refers to data collected by the project, while the term secondary data refers to data which were gathered by another entity. Secondary data included those obtained from DHS and MICS data sets. Finally, each indicator was further classified as to whether it was referring to a project input, process, output, outcome or impact.

## RESULTS

### Location of included projects

The assessments included in our analysis were for projects from various regions of the world (**Table 1**). One of the studies included data from 28 African countries, and another had data from four African countries. All other studies focused on one country or a smaller sub–population within that country as shown in **Table 1**.

**Table 1:** Geographical location of reports containing equity analyses

Geographical region	Number of studies
Africa	19
Southeast Asia	14
Americas	8
Western Pacific	1
Total	42

### Kinds of data used in the assessments

The data utilized in 37 of the 42 projects including equity analyses collected specifically by the project within the project’s geographic area. However, five analyses exclusively utilized data from DHS and MICS surveys, and two utilized both project–level data collected for assessment of the project and also publicly available national data.

### Criteria through which equity effects were assessed

Across the 42 projects included in our analysis, 82 equity indicators — for example coverage of prenatal home visits analyzed across household income categories (Callaghan–Koru, 2013; reference [S15] in **Online Supplementary Document(Online Supplementary Document)**) — were identified. Equity was measured by comparing changes in health program characteristics or health status over time for more disadvantaged groups with changes in the identical indicators for more advantaged groups. **Table 2** summarizes the criteria by which disadvantaged groups were distinguished from more advantaged groups.

**Table 2:** Equity indicators used in the assessments included in the analysis

Equity criterion	Number of assessments in which the indicator was used*	Comparable USAID PROGRESS Indicators
**Socioeconomic status (SES):**		
Household income categories	45	Wealth
Household assets (production, other assets such as savings)	5	Wealth
Maternal education	9	Gender
Social standing (ethnicity, caste, religion, parent marital status)	8	Ethnicity
Parent occupation	1	Wealth
**Other:**		
Geographic location of residence (urban vs rural)	24	Geography
Child’s sex	3	Gender
Nutritional status	4	Wealth
Maternal age	2	Age
Country–level Human Development Index (HDI)	1	Wealth

*The column total is 82 since many of the assessments in our review included more than one equity indicator.

We grouped several equity indicators under a category we refer to as socioeconomic status (SES). These included income categories, maternal education, and household characteristics. By far, the most common indicator for assessing equity was a measure of wealth, often based on household income, household assets, household size, or maternal earnings. Other SES equity indicators included in the analysis were agricultural production by heads of household and specific assets present in the household such as a working toilet, running water, or a refuse collection system. Other SES criteria included the ethnic group of the family, religion, marital status of child’s parents, occupation of the parents, and demographic characteristics such as maternal age. These equity indicators aligned well with those identified by the USAID PROGRESS report (shown in the right–hand column of **Table 2**); the only PROGRESS category that was not identified in our analysis was religion [27].

### Assessments of equity of inputs

After careful analysis and discussion among co–authors and colleagues, we determined that no projects that we included in our data set explicitly analyzed or reported inputs from an equity perspective. The dearth of input–related efforts in project design, implementation, and evaluation is concerning and is noted as an area where further work is needed.

### Assessments of equity using processes

A number of the assessments included in our review measured process indicators through an equity lens, as shown in **Table 3** (references in Tables 3–6, are prefixed with an S and appear in **Online Supplementary Document(Online Supplementary Document)**). Two–thirds (10/13) of the measurements of equity involving process indicators concerned whether the household had received a home visit from a health worker or had contact with the health system. Eleven out of 13 of the measurements yielded a pro–equitable result, and the remaining two yielded an equitable result. Thus, for the process indicators in the assessments selected for analysis, equity had been achieved in all cases and a pro–equity result is observed in almost all. The findings for this portion of the log frame consistently support the equitable nature of home visiting practices, a central feature of many CBPHC projects, as also discussed in the in this supplement that directly address the effectiveness of CBPHC in improving MNCH [30–32]. Many of these home visits either implicitly or explicitly included promotion and support of breastfeeding, which has also been noted in the literature as an intervention that can be supported equitably through community–based approaches with multiple benefits to MNCH.

**Table 3:** Assessments of equity effects of CBPHC projects using process indicators*

Process indicator	Equity criterion	Outcome	Reference
Postnatal home visit	Household income	Equitable	Callaghan–Koru 2013 [S15]
Home visit during pregnancy	Household income	Equitable	Callaghan–Koru 2013 [S15]
Azythromycin distribution to entire communities for trachoma	Household assets	Pro–equitable	Cumberland 2008 [S19]
CHW visit to caregivers within the past year	Urban vs rural	Pro–equitable	Litrell 2013 [S25]
Caregivers report of CHWs working in community	Urban vs rural	Pro–equitable	Litrell 2013 [S25]
Prenatal home visit	Household income	Pro–equitable	Baqui 2008 [S8]
Number of home visits	Urban vs rural	Pro–equitable	Perry 2006 [S35]
Antenatal home visit	Household income	Pro–equitable	Baqui 2008 [S8]
At least one home visit during pregnancy	Household income	Pro–equitable	Callaghan–Koru 2013 [S15]
Two or more home visits during pregnancy	Household income	Pro–equitable	Callaghan–Koru 2013 [S15]
Home visits to support breastfeeding	Household income, maternal education	Pro–equitable	Coutinho 2005 [S17]
Child ill and CHW called to come to the home	Household income	Pro–equitable	Siekmans 2013 [S38]
At least one ANC visit in home	Household income	Pro–equitable	Nonyane 2015 [S32]

*References which are prefixed with an S appear in Appendix S1 of the **online supplementary document(Online Supplementary Document)**.

### Assessments of equity of outputs

The assessments of equity using output indicators are listed in **Table 4**. Two–thirds (4/6) of the six equity assessments using output indicators among the projects selected for our analysis concerned the utilization of specific services or the expected immediate output of an intervention. Half (3/6) of these equity assessments used household income as the equity criterion. The number of assessments is too small to make major generalizations from, but the indicators demonstrating a pro–equity effect in the output category focus on access to health services (either in a facility or in the home). Indicators that demonstrated an inequitable effect were both from the same study and related to the hygienic practices across several equity dimensions.

**Table 4:** Assessments of equity effects of CBPHC projects using output indicators*

Output indicator	Equity criterion	Outcome	Reference
Food hygiene score in relation to cleanliness score	Household income	Inequitable	Ahmed 1993 [S1]
Food hygiene score in relation to diarrhea prevalence	Maternal education, nutritional status	Inequitable	Ahmed 1993 [S1]
Utilization of ambulatory care facility	Urban vs rural	Pro–equitable	Perry 2006 [S35]
Number of hospital admissions	Urban vs rural	Pro–equitable	Perry 2006 [S35]
Child with fever treated within 24 h	Household income	Pro–equitable	Siekmans 2013 [S38]
Essential newborn practices performed	Household income	Pro–equitable	Baqui 2008 [S8]

*References which are prefixed with an S appear in Appendix S1 of the **online supplementary document(Online Supplementary Document)**.

### Assessments of equity of outcomes

**Table 5** below lists the equity assessments carried out using outcome indicators. Many relate to knowledge and behavior change related to breastfeeding or to the population coverage level of an intervention. Of the 35 measurement carried out, only 14% (5/35) yielded an inequitable result; 11% (4/35) yielded an equitable result, and the rest (74%) yielded a pro–equitable result. Inequitable indicators included several interventions requiring significant equipment or knowledge such as vaccine coverage and antenatal and delivery care. Some indicators — such as ITN coverage, availability, and use — showed mixed results across different studies, with some having equitable results across household income categories or urban and rural settings and others not. Equitable and pro–equitable programs commonly focused on equitable behaviors such as breastfeeding and newborn and child health practices that can be implemented in the home without complex or expensive supplies or knowledge.

**Table 5:** Assessments of equity effects of CBPHC projects using outcome indicators*

Outcome indicator	Equity criterion	Outcome	Reference
Understanding of overall cleanliness	Maternal education	Inequitable	Ahmed 1993 [S1]
Coverage of antenatal and delivery care	Household income	Inequitable	Bryce 2008 [S14]
EPI immunization coverage	Household income	Inequitable	Webster 2005 [S42]
ITN coverage	Household income	Inequitable	Webster 2005 [S42]
Coverage of any type of bed net (ITN or other)	Household income	Inequitable	Webster 2005 [S42]
Health service coverage	Child’s sex	Equitable	Bryce 2008 [S14]
Nothing applied to umbilical cord by mother after birth	Household income	Equitable	Nonyane 2015 [S32]
Child with diarrhea treated with ORS or zinc	Household income	Equitable	Littrell 2013 [S25]
Awareness of support group in community	Household income	Equitable	Callaghan–Koru 2013 [S15]
Exclusive breastfeeding	Urban vs rural	Pro–equitable	Crookston 2000 [S18]
Exclusive breastfeeding from birth to 6m	Household income	Pro–equitable	Coutinho 2005 [S17]
Breastfeeding initiation within first hour of life	Urban vs rural	Pro–equitable	Crookston 2000 [S17]
Breastfeeding initiation within first hour of life	Household income	Pro–equitable	Nonyane 2015 [S32]
Knowledge of family planning methods	Urban vs rural	Pro–equitable	Debpuur 2002 [S20]
Knowledge and use of family planning	Maternal education, social standing	Pro–equitable	Awooner–Williams 2004 [S5]
Recognition of at least 3 danger signs in newborns	Household income	Pro–equitable	Nonyane 2015 [S32]
Child with fever treated with artemether–lumefantrine within 48 hours	Household income	Pro–equitable	Siekmans 2013 [S38]
Acute respiratory infection treatment rate	Household income	Pro–equitable	Mercer 2004 [S28]
Any bed net available	Household income	Pro–equitable	Skarbinski 2007 [S39]
Measles vaccination rate	Household income	Pro–equitable	Mercer 2004 [S28]
Immunization coverage	Household income	Pro–equitable	Bawah 2006 [S10]
ITN in home	Household income	Pro–equitable	Skarbinski 2007 [S39]
ITN coverage	Urban vs rural	Pro–equitable	Grabowsky 2005 [S23]
ITN coverage	Household income	Pro–equitable	Grabowsky 2005 [S23]
ITN coverage	Household income	Pro–equitable	Noor 2007 [S33]
Immediate drying	Household income	Pro–equitable	Nonyane 2015 [S32]
Postnatal care coverage	Maternal education, household income, social standing, household assets	Pro–equitable	Awooner–Williams 2004 [S5]
Children sleeping under ITNs	Household income	Pro–equitable	Noor 2007 [S33]
Attended delivery	Maternal education, household income, social standing, household assets	Pro–equitable	Awooner–Williams 2004 [S5]
Antenatal care	Maternal education, household income, social standing, household assets	Pro–equitable	Awooner–Williams 2004 [S5]
Antenatal care coverage	Household income	Pro–equitable	Baqui 2008 [S8]

*References which are prefixed with an S appear in Appendix S1 of the **online supplementary document(Online Supplementary Document)**.

### Assessments of equity of health impact

Finally, **Table 6** lists the assessments of health equity that were carried out for health impact–related indicators (nutritional status, morbidity or mortality). Of the 28 projects that included an equity assessment of health impact, 20 were based on a measure of mortality; four were based on a measure of morbidity and four on a measure of nutritional status. Overall, 23 of the 28 assessments demonstrated pro–equitable results and one yielded an equitable result. Only four of the 28 yielded an inequitable result.

**Table 6:** Assessments of equity of CBPHC projects using impact indicators*

Impact indicator	Equity criterion	Outcome	Reference
Neonatal morality rate	Household income	Inequitable	Razzaque 2007 [S36]
Under–5 mortality rate	Urban vs rural	Inequitable	Bryce 2008 [S14]
Under–5 mortality rate	Household income	Inequitable	Razzaque 2007 [S36]
Child (age 6–59 months) mortality rate	Social standing, child’s sex	Inequitable	Bishai 2005 [S12]
Tetanus neonatorum mortality rate	Urban vs rural	Equitable	Newell 1966 [S31]
Diarrhea prevalence in children 0–36 months of age	Urban vs rural	Pro–equitable	Barreto 2007 [S9]
Diarrhea prevalence in children 0–18 months of age	Nutritional status	Pro–equitable	Ahmed 1993 [S1]
Diarrhea prevalence in children 0–36 months of age	Urban vs rural	Pro–equitable	Barreto 2007 [S9]
Undernutrition prevalence	Nutritional status	Pro–equitable	Mustaphi 2005 [S30]
Child nutrition status (qualitative data)	Nutritional status	Pro–equitable	McNelly 1998 [S29]
Perinatal mortality rate	Urban vs rural	Pro–equitable	Bang 2005 [S7]
Perinatal mortality rate	Urban vs rural	Pro–equitable	Bang 1999 [S6]
Neonatal mortality rate	Urban vs rural	Pro–equitable	ASHA–India 2008 [S4]
Neonatal mortality rate	Urban vs rural	Pro–equitable	Bang 1999 [S6]
Infant mortality rate	Maternal education, child’s sex	Pro–equitable	Fegan 2007 [S21]
Infant mortality rate	Urban vs rural	Pro–equitable	Asha–India 2008 [S4]
Infant mortality rate	Social standing, parental occupation	Pro–equitable	Bang 1999 [S6]
Infant mortality rate	Household income	Pro–equitable	Bhuiya 2002 [S11]
Infant mortality rate	Household assets, maternal education	Pro–equitable	Bang 2005 [S7]
Infant mortality rate	Human development index	Pro–equitable	Aquino 2009 [S2]
Infant mortality rate	Household income	Pro–equitable	Mercer 2004 [S28]
Infant, 1–4 years, and under–5 mortality rates	Household income	Pro–equitable	Mercer 2004 [S28]
Under–5 mortality rate	Household income	Pro–equitable	Sepulveda 2006 [S37]
Under–5 mortality rate	Urban vs rural, household income	Pro–equitable	Asha–India 2008 [S4]
Under–5 mortality rate	Urban vs rural	Pro–equitable	Perry 2006 [S35]
Under–5 mortality rate	Household income	Pro–equitable	Bryce 2008 [S14]
Under–5 mortality rate	Urban vs rural	Pro–equitable	Asha–India 2008 [S4]

*References which are prefixed with an S appear in Appendix S1 of the **online supplementary document(Online Supplementary Document)**.

### Overall summary of equity effects using household wealth as the equity criterion

We have summarized all the findings reported above in which household income was the equity criterion (**Table 7**). Overall, 75% (33/44) of these effects were pro–equitable outcome, 9% were equitable outcome, and only 16% (7/44) yielded an inequitable effect.

**Table 7:** Summary of assessments of equity using socio–economic status or household wealth quintile as the equity criterion

Type of indicator	Effect on equity			
**Inequitable**	**Equitable**	**Pro–equitable**	**Total**
Input	0	0	0	0
Process	0	2	7	9
Output	1	0	2	3
Outcome	4	2	18	24
Impact	2	0	6	8
Total	7	4	33	44

### Overall summary of all equity effects

Finally, we have summarized equity effects in **Table 8**. Overall, 78% (64/82) of the equity assessments carried out yielded a pro–equitable outcomes; 9% (7/82) yielded an equitable outcomes, and only 13% (11/82) yielded an inequitable outcome.

**Table 8:** Summary of all assessments of equity

Type of indicator	Effect on equity			
**Inequitable**	**Equitable**	**Pro–equitable**	**Total**
Input	0	0	0	0
Process	0	2	11	13
Output	2	0	4	6
Outcome	5	4	26	35
Impact	4	1	23	28
Total	11	7	64	82

While in–depth analysis of the impact of packages of interventions was not the focus of this paper (another paper in this series [33] addresses this strategy in general – not limited to equity), we reviewed which projects constituted a single intervention vs a package of interventions. Of the 42 projects, 11 (26%) included a single intervention while eight (19%) included 2 interventions, and 23 (55%) of projects had a package of three or more services. We could not identify any clear patterns between the number of interventions and how equitable the findings were; the only clear pattern was that, in general, all interventions and equity dimensions within any particularly project tended to be the same in terms of equity outcomes (eg, all of the findings for Ahmed 1993 were inequitable).

Of the 42 projects that conducted an equity analysis, we also reviewed which ones analyzed more one or more dimensions of equity. 27 (64%) included an analysis for only one dimension of equity while nine (21%) included two dimensions of equity, and only six (14%) included three or more dimensions of equity. We also did not identify any obvious patterns among the small groups of projects in each of these categories. Household income as part of SES was by far the most common dimension of equity, and was utilized across all of these categories followed closely by comparing urban vs rural populations. The projects with inequitable findings included a number of SES analyses and also child gender and an urban vs rural comparison.

## DISCUSSION

We have carried out an equity analysis of the projects in our review that contained evidence regarding the equity effects of CBPHC in improving child health. Out of the 546 assessments related to child health in our data set, 42 measured equity effects. Of the 82 measurements of equity effects in these 42 projects, 87% of these measurements indicated that the equity effect was either equitable (in which the disadvantage group benefitted to the same degree as the more advantaged group) or pro–equitable (in which the disadvantaged group benefitted more). Of the 42 articles in our review, 15 of them (36%) measured two or more equity dimensions and 31 articles (74%) measured equity across two or more interventions. These findings provide strong evidence of the capacity of CPBHC to reduce inequities in the delivery of child health services and in child health outcomes. Thus, these findings are consistent with the assertion that CBPHC has the potential to reduce inequities in child health in low–income settings where health facilities alone would be highly unlikely to reduce existing inequities since, in fact, it is well–known that health facility utilization in low–income settings is highly inequitable, as explained further below.

The counter–argument to this assertion is that expansion of the number of facilities and improvements in facility–based care will eventually reduce inequities in child health. This may be possible in the very long term, but there is no evidence at present that we are aware of demonstrating that expanding or improving facility–based services as an isolated strategy reduces inequities in the delivery of child health services or in child health status. For the near term, resources will continue to be highly constrained in low–income countries and major geographic [34], social and financial barriers will continue to exist in accessing facility–based care. Therefore, our findings indicate that strong expansion of CBPHC will be required to reduce inequities in child health.

A case example from Brazil of equity effects of CBPHC on improving child health (an article selected from our database) that serves as an example of the potential pro–equity effects of combining community–based approaches with political will and investment, a national strategy, and a long–term commitment.

Aquino et al., 2008 (reference [S12] in **Online Supplementary Document(Online Supplementary Document)**) analyzed the effects of expanding Brazil’s Family Health Program (FHP) coverage on infant mortality. They identified that the effect of the FHP program was greatest in terms of decreasing infant mortality in municipalities where infant mortality was highest and the human development index was lowest at the beginning of the study period. The FHP program used a family–centered approach to provide a range of services at the community level, including promotion of breastfeeding, prenatal care, immunizations, and management of diarrhea. The team of health workers, in addition to physicians and nurses as well as oral health professionals, includes CHWs (called Community Health Agents) who visited every home on a monthly basis. This national program has brought Brazil global recognition for its efforts to reduce health inequities for the general population and for children in particular (including inequities of childhood nutritional status). A high level of political will has been necessary in order to implement the scale and depth of this program at the national level.

### Explaining the pro–equity effects of community–based primary health care

Most CBPHC projects are designed to reach every household with health education and information about how to access outreach services (if not to actually provide services including curative care), and outreach services are generally distributed more evenly throughout target populations than facility–based services [35]. Meanwhile, some countries, such as Peru, where great investment in health facilities has taken place—including expansion of community health centers—these efforts have resulted in only very small improvements on equitable utilization of health facilities [36].

Research on the equity of facility utilization in low–income settings is limited; more evidence is available for high–income settings in the Americas, Europe, and Asia. In LMIC settings, health facilities tend to be few and far between, often expensive from the perspective of the poor, and lacking high quality of care, including provision of care that is seen by certain sub–groups as disrespectful [36–38]. Factors such as education level, income, and urban and rural residence play key roles in determining whether someone is more or less likely to seek care at a health facility [36,37]. Thus, the effort and resources that patients and their families have to expend to reach a health facility and the uncertain return on that family’s investment contributes to low utilization of facility–based services.

The challenge of providing interventions that are often only available in health facilities – or require infrastructure and skills difficult to deploy in communities outside of facilities—is significant as well. A growing literature, including but also going beyond the database used in this study, points to inequitable usage of health facilities in terms of the SES and urban/rural characteristics of users [23,34,39].

The need for alternative approaches beyond health facilities to achieve equity in and in fact universal coverage for child health are the following: (i) there is an exponential decline in the utilization of health facilities with increasing distance to the health facility (particularly more than 5 km or 1 hour walk away) [35], and (ii) there is a need for available and affordable public transportation in order to reach health facilities, which is often absent [33,39]. What is lacking from the literature are in–depth assessments of equity of health care utilization in terms of distance from a health facility and the effect of distance from health facilities on health status, taking into account also whether community–based care is available to those further away from those facilities.

Strong community–based programs can encourage facility utilization across income strata as can vouchers provided at the community level for specific services, such as antenatal care, to reduce resource barriers to seeking care [40]. The available evidence suggests that CBPHC approaches that reach all households can be more equitable than solely facility–based approaches in terms of coverage of a number of key primary health care services, particularly for vulnerable populations and those who live further away from facilities, who are also usually more disadvantaged in terms of SES [20,41–43].

There are several assessments that directly compare the degree to which CBPHC approaches as opposed to other approaches improve the health of the poorest segment of the project population compared to that of the better off segment. It makes sense that home–centered, low–resource interventions like breastfeeding promotion and distribution of ITNs would be able to achieve high levels of equity through community–based approaches that often include direct contact with all households [7]. In addition, some of the most promising strategies to improve health equity focus on strengthening community outreach, using CHWs and other lay workers, along with market–driven options such as minimizing or removing user fees and engaging the private sector [3,44,45].

Approaches that make it possible for health workers to reach all households – or at least to reach outreach points that are relatively evenly distributed throughout the project population and close to homes – are inherently more likely to achieve favorable equity effects than facility–based approaches. However, a number of other equity–relevant factors including education, child’s sex, ethnicity [46], and urban vs rural contexts [47] cannot be overlooked even within such a strong outreach approach [48]. Health programs in high–mortality, resource–constrained settings lack the capacity to build and operate facilities within easy reach of all who could need to use them – particularly in low–density rural areas. Thus, the decentralization of services and utilization of innovative and proven strategies to support the coverage, quality, and sustainability of those services is essential for achieving health equity.

While the focus of this review is on low–income countries, inequities are also prevalent in higher–income countries as well. Even where more resources are available to address such issues, political will is needed to direct those resources in ways that decrease inequities. An example of progress and success in the arena of health equity is Japan’s national policies to provide equitable educational opportunities as well as access to health services without financial barriers [49]. Globally, but particularly in low–income countries, much work remains to be done to make this kind of progress a reality for all populations. In addition to our public health–specific tools and approaches, more comprehensive community development and empowerment frameworks, such as the CHOICE (**C**apacity–building, **H**uman rights, **O**rganizational sustainability, **I**nstitutional accountability, **C**ontribution, and **E**nabling environment) framework [50], can help to frame issues of health equity and provide additional entry points for understanding and addressing them. As Victora et al. note [51], just using the data available and recognizing patterns in inequities is not enough; political will and deliberate design and attention to the causes of inequities in programs for child health are necessary to achieve substantial decreases in child mortality among the most disadvantaged sub–populations where the mortality rates are the greatest.

Community–based approaches can reach those furthest from health facilities and can rapidly expand population coverage of key interventions, so these findings are not surprising. These findings stand in stark contrast to the commonly observed finding that utilization of primary health care facilities is inequitable because those in the lower income quintiles are less likely to obtain services there [52,53]. To our knowledge, this is the first comprehensive review in the peer–reviewed literature summarizing the equity effects of CBPHC in improving child health.

### Limitations of our study

This study has several limitations that we want to make explicit. First, we have not further disaggregated the articles based on how strong the equity effect is. Second, some of the 42 assessments qualifying for our analysis are efficacy studies conducted within community settings in which ideal conditions were present for project implementation. Therefore, we must be careful about generalizing these findings to everyday practice settings. But, that said, it still remains true that strong pro–equity effects are achievable through CBPHC. An analysis of the quality of the data included in the 42 assessments included in our review was beyond the scope of this article. Finally, although a thorough search has been conducted that covers articles published over the past six decades through the end of 2015, we know that there are likely to be more recent articles published since that time that are relevant to this analysis.

We have worked to be clear in our language, conservative in our claims, and yet optimistic about the role of community–based approaches to continue to help bolster health equity for children in disadvantaged populations around the world.

## CONCLUSIONS

Based on the finding that the services provided by CBPHC projects generally reach most or all households and are readily accessible throughout the project population, CBPHC projects are inherently more likely to achieve pro–equity effects than projects that strengthen services only at facilities. The decentralization of service provision and management and the utilization of community–level workers are important for reducing inequities in national programs of countries where the risk of child mortality is high. Equity assessments need to become a standard feature of MNCH programming.
