# Supplementary material for: Comprehensive review of the evidence regarding the effectiveness of community–based primary health care in improving maternal, neonatal and child health: 5. equity effects for neonates and children
Source: J Glob Health. 2017 Jun 29;7(1):010905. doi: 10.7189/jogh.07.010905 (PMC5491949; doi:10.7189/jogh.07.010905)
Supplement: Online Supplementary Document [file jogh-07-010905-s001.pdf]

Online Supplementary Document

Schlieff et al. Comprehensive review of the evidence regarding the effectiveness of Community–Based Primary Health Care in improving maternal, neonatal and child health: 5. equity effects for neonates and children

J Glob Health 2017;7:010905

References of Assessments Included in the Equity Analysis

S1 Ahmed, N. U., Zeitlin, M. F., Beiser, A. S., Super, C. M., & Gershoff, S. N. (1993). A longitudinal study of the impact of behavioural change intervention on cleanliness, diarrhoeal morbidity and growth of children in rural Bangladesh. Soc Sci Med, 37(2), 159-171. doi:http://dx.doi.org/10.1016/0277-9536(93)90452-a

S2 Aquino, R., de Oliveira, N. F., & Barreto, M. L. (2009). Impact of the family health program on infant mortality in Brazilian municipalities. Am J Public Health, 99(1), 87-93. doi:10.2105/AJPH.2007.127480

S3 Arifeen, S. E., Hoque, D. M., Akter, T., Rahman, M., Hoque, M. E., Begum, K., . . . Black, R. E. (2009). Effect of the Integrated Management of Childhood Illness strategy on childhood mortality and nutrition in a rural area in Bangladesh: a cluster randomised trial. Lancet, 374(9687), 393-403. doi:10.1016/S0140-6736(09)60828-X

S4 Asha/India (2008). Overview

S5 Awoonor-Williams, J. K., Feinglass, E. S., Tobey, R., Vaughan-Smith, M. N., Nyonator, F. K., & Jones, T. C. (2004). Bridging the gap between evidence-based innovation and national health-sector reform in Ghana. Stud Fam Plann, 35(3), 161-177. doi:http://dx.doi.org/10.1111/j.1728-4465.2004.00020.x

S6 Bang, A. T., Bang, R. A., Baitule, S. B., Reddy, M. H., & Deshmukh, M. D. (1999). Effect of home-based neonatal care and management of sepsis on neonatal mortality: field trial in rural India. Lancet, 354(9194), 1955-1961. doi:10.1016/S0140-6736(99)03046-9

S7 Bang, A. T., Reddy, H. M., Deshmukh, M. D., Baitule, S. B., & Bang, R. A. (2005). Neonatal and infant mortality in the ten years (1993 to 2003) of the Gadchiroli field trial: effect of home-based neonatal care. J Perinatol, 25 Suppl 1, S92-107. doi:10.1038/sj.jp.7211277

S8 Baqui, A. H., Rosecrans, A. M., Williams, E. K., Agrawal, P. K., Ahmed, S., Darmstadt, G. L., . . . Santosham, M. (2008). NGO facilitation of a government community-based maternal and neonatal health programme in rural India: improvements in equity. Health Policy Plan, 23(4), 234-243. doi:10.1093/heapol/czn012

S9 Barreto, M. L., Genser, B., Strina, A., Teixeira, M. G., Assis, A. M., Rego, R. F., . . . Cairncross, S. (2007). Effect of city-wide sanitation programme on reduction in rate of childhood diarrhoea in northeast Brazil: assessment by two cohort studies. Lancet, 370(9599), 1622-1628. doi:10.1016/S0140-6736(07)61638-9

S10 Bawah, A. A., Philips, J. F., Adjuik, M., Vaughan-Smith, M., Macleod, B., & Binka, F. N. (2006). The impact of immunization on the association between poverty and child survival: evidence from Kassena-Nankana district of northern Ghana. Population Council Working Papers 2/8. New York, NY

S11 Bhuiya, A., & Chowdhury, M. (2002). Beneficial effects of a woman-focused development programme on child survival: evidence from rural Bangladesh. Soc Sci Med, 55(9), 1553-1560. doi:http://dx.doi.org/10.1016/s0277-9536(01)00287-8

S12 Bishai, D., Kumar, K. C. S., Waters, H., Koenig, M., Katz, J., Khatry, S. K., & West, K. P., Jr. (2005). The impact of vitamin A supplementation on mortality inequalities among children in Nepal. Health Policy Plan, 20(1), 60-66. doi:10.1093/heapol/czi007

S13 Bojang, K. A., Akor, F., Conteh, L., Webb, E., Bittaye, O., Conway, D. J., . . . Greenwood, B. (2011). Two strategies for the delivery of IPTc in an area of seasonal malaria transmission in the Gambia: a randomised controlled trial. PLoS Med, 8(2), e1000409. doi:10.1371/journal.pmed.1000409

S14 Bryce, J., Gilroy, K., Jones, G., Hazel, E., Black, R. E., & Victora, C. G. (2008). The Retrospective Evaluation of ACSD: Cross-site analyses and conclusions

S15 Callaghan-Koru, J. A., Nonyane, B. A., Guenther, T., Sitrin, D., Ligowe, R., Chimbalanga, E., . . . Baqui, A. H. (2013). Contribution of community-based newborn health promotion to reducing inequities in healthy newborn care practices and knowledge: evidence of improvement from a three-district pilot program in Malawi. BMC Public Health, 13, 1052. doi:10.1186/1471-2458-13-1052

S16 Cesar, J. A., Goncalves, T. S., Neumann, N. A., Oliveira Filho, J. A., & Diziekaniak, A. C. (2005). [Child health in poor areas of North and Northeast Brazil: a comparison of areas covered by the Children's Mission and control areas]. Cad Saude Publica, 21(6), 1845-1855. doi:/S0102-311X2005000600034

S17 Coutinho, S. B., de Lira, P. I., de Carvalho Lima, M., & Ashworth, A. (2005). Comparison of the effect of two systems for the promotion of exclusive breastfeeding. Lancet, 366(9491), 1094-1100. doi:10.1016/S0140-6736(05)67421-1

S18 Crookston, B. T., Dearden, K. A., Chan, K., Chan, T., & Stoker, D. D. (2007). Buddhist nuns on the move: an innovative approach to improving breastfeeding practices in Cambodia. Matern Child Nutr, 3(1), 10-24. doi:10.1111/j.1740-8709.2007.00074.x

S19 Cumberland, P., Edwards, T., Hailu, G., Harding-Esch, E., Andreasen, A., Mabey, D., & Todd, J. (2008). The impact of community level treatment and preventative interventions on trachoma prevalence in rural Ethiopia. Int J Epidemiol, 37(3), 549-558. doi:10.1093/ije/dyn045

S20 Debpuur, C., Phillips, J. F., Jackson, E. F., Nazzar, A., Ngom, P., & Binka, F. N. (2002). The impact of the Navrongo Project on contraceptive knowledge and use, reproductive preferences, and fertility. Stud Fam Plann, 33(2), 141-164. doi:http://dx.doi.org/10.1111/j.1728-4465.2002.00141.x

S21 Fegan, G. W., Noor, A. M., Akhwale, W. S., Cousens, S., & Snow, R. W. (2007). Effect of expanded insecticide-treated bednet coverage on child survival in rural Kenya: a longitudinal study. Lancet, 370(9592), 1035-1039. doi:10.1016/S0140-6736(07)61477-9

S22 Fernald, L. C., Gertler, P. J., & Neufeld, L. M. (2008). Role of cash in conditional cash transfer programmes for child health, growth, and development: an analysis of Mexico's Oportunidades. Lancet, 371(9615), 828-837. doi:10.1016/S0140-6736(08)60382-7

S23 Grabowsky, M., Farrell, N., Hawley, W., Chimumbwa, J., Hoyer, S., Wolkon, A., & Selanikio, J. (2005). Integrating insecticide-treated bednets into a measles vaccination campaign achieves high, rapid and equitable coverage with direct and voucher-based methods. Trop Med Int Health, 10(11), 1151-1160. doi:10.1111/j.1365-3156.2005.01502.x

S24 Kumar, V., Mohanty, S., Kumar, A., Misra, R. P., Santosham, M., Awasthi, S., . . . Saksham Study, G. (2008). Effect of community-based behaviour change management on neonatal mortality in Shivgarh, Uttar Pradesh, India: a cluster-randomised controlled trial. Lancet, 372(9644), 1151-1162. doi:10.1016/S0140-6736(08)61483-X

S25 Littrell, M., Moukam, L. V., Libite, R., Youmba, J. C., & Baugh, G. (2013). Narrowing the treatment gap with equitable access: mid-term outcomes of a community case management program in Cameroon. Health Policy Plan, 28(7), 705-716. doi:10.1093/heapol/czs110

S26 Lutter, C. K., Rodriguez, A., Fuenmayor, G., Avila, L., Sempertegui, F., & Escobar, J. (2008). Growth and micronutrient status in children receiving a fortified complementary food. J Nutr, 138(2), 379-388.

S27 Mbonye, A. K., Hansen, K. S., Bygbjerg, I. C., & Magnussen, P. (2008). Intermittent preventive treatment of malaria in pregnancy: the incremental cost-effectiveness of a new delivery system in Uganda. Trans R Soc Trop Med Hyg, 102(7), 685-693. doi:10.1016/j.trstmh.2008.04.016

S28 Mercer, A., Khan, M. H., Daulatuzzaman, M., & Reid, J. (2004). Effectiveness of an NGO primary health care programme in rural Bangladesh: evidence from the management information system. Health Policy Plan, 19(4), 187-198. doi:http://dx.doi.org/10.1093/heapol/czh024

S29 MkNelly, B., & Dunford, C. (1998). Impact of credit with education on mother and their young children's nutrition: Lower Pra rural bank credit with education program in Ghana. Freedom from hunger Research Paper no. 4

S30 Mustaphi, P., & Dobe, M. (2005). Positive deviance--the West Bengal experience. Indian J Public Health, 49(4), 207-213.

S31 Newell, K. W., Duenas Lehmann, A., LeBlanc, D. R., & Garces Osorio, N. (1966). The use of toxoid for the prevention of tetanus neonatorum. Final report of a double-blind controlled field trial. Bull World Health Organ, 35(6), 863-871.

S32 Nonyane, B. A., Kc, A., Callaghan-Koru, J. A., Guenther, T., Sitrin, D., Syed, U., . . . Baqui, A. H. (2015). Equity improvements in maternal and newborn care indicators: results from the Bardiya district of Nepal. Health Policy Plan. doi:10.1093/heapol/czv077

S33 Noor, A. M., Amin, A. A., Akhwale, W. S., & Snow, R. W. (2007). Increasing coverage and decreasing inequity in insecticide-treated bed net use among rural Kenyan children. PLoS Med, 4(8), e255. doi:10.1371/journal.pmed.0040255

S34 Nyonator, F., Jones, T. C., Miller, R. A., Phillips, J. F., & Awoonor-Williams, J. K. (2004-2005). Guiding the Ghana community-based health planning and services approach to scaling up with qualitative systems appraisal. Int Quaterly of Community Health Education, 23(3), 189-213. doi:http://dx.doi.org/10.2190/ngm3-fydt-5827-ml1p

S35 Perry, H., Cayemittes, M., Philippe, F., Dowell, D., Dortonne, J. R., Menager, H., . . . Berggren, G. (2006). Reducing under-five mortality through Hopital Albert Schweitzer's integrated system in Haiti. Health Policy Plan, 21(3), 217-230. doi:10.1093/heapol/czl005

S36 Razzaque, A., Streatfield, P. K., & Gwatkin, D. R. (2007). Does health intervention improve socioeconomic inequalities of neonatal, infant and child mortality? Evidence from Matlab, Bangladesh. Int J Equity Health, 6, 4. doi:10.1186/1475-9276-6-4

S37 Sepulveda, J., Bustreo, F., Tapia, R., Rivera, J., Lozano, R., Olaiz, G., . . . Valdespino, J. L. (2006). Improvement of child survival in Mexico: the diagonal approach. Lancet, 368(9551), 2017-2027. doi:10.1016/S0140-6736(06)69569-X

Siekmans, K., Sohani, S., Kisia, J., Kiilu, K., Wamalwa, E., Nelima, F., . . . Ngindu, A. (2013). Community case management of malaria: a pro-poor intervention in rural Kenya. Int Health, 5(3), 196-204. doi:10.1093/inthealth/iht017

S38 Skarbinski, J., Massaga, J. J., Rowe, A. K., & Kachur, S. P. (2007). Distribution of free untreated bednets bundled with insecticide via an integrated child health campaign in Lindi Region, Tanzania: lessons for future campaigns. Am J Trop Med Hyg, 76(6), 1100-1106.

S39 Tiono, A. B., Kabore, Y., Traore, A., Convelbo, N., Pagnoni, F., & Sirima, S. B. (2008). Implementation of Home based management of malaria in children reduces the work load for peripheral health facilities in a rural district of Burkina Faso. Malar J, 7, 201. doi:10.1186/1475-2875-7-201

S40 Wafula, E. M., Kinyanjui, M. M., Nyabola, L., & Tenambergen, E. D. (2000). Effect of improved stoves on prevalence of acute respiration infection and conjunctivitis among children and women in a rural community in Kenya. East Afr Med J, 77(1), 37-41. doi:http://dx.doi.org/10.4314/eamj.v77i1.46379

S41 Webster, J., Lines, J., Bruce, J., Armstrong Schellenberg, J. R., & Hanson, K. (2005). Which delivery systems reach the poor? A review of equity of coverage of ever-treated nets, never-treated nets, and immunisation to reduce child mortality in Africa. Lancet Infect Dis, 5(11), 709-717. doi:10.1016/S1473-3099(05)70269-3
